# Case Report: Pirfenidone in the Treatment of Post-COVID-19 Pulmonary Fibrosis

**DOI:** 10.3389/fmed.2022.925703

**Published:** 2022-06-06

**Authors:** Xianglin Zhou, Danhui Yang, Xianglong Kong, Chengli Wei, Siqi LvQiu, Lin Wang, Yongkang Lin, Zhilan Yin, Zhiguo Zhou, Hong Luo

**Affiliations:** ^1^Department of Pulmonary and Critical Care Medicine, the Second Xiangya Hospital, Central South University, Changsha, China; ^2^Research Unit of Respiratory Disease, Central South University, Changsha, China; ^3^Hunan Diagnosis and Treatment Center of Respiratory Disease, Changsha, China; ^4^Respiratory Medicine, The First Hospital of Changsha, Changsha, China; ^5^Department of Radiology, The Second Xiangya Hospital, Central South University, Changsha, China

**Keywords:** pulmonary fibrosis, post-COVID-19, pirfenidone, long COVID, sequelae

## Abstract

**Background:**

Pulmonary fibrosis is one of the sequelae of the COVID-19, which seriously affects the quality of life of survivors. Currently, there are no optimal evidence based guidelines targeting this population.

**Case Presentation:**

We report a 66-year-old female patient without underlying comorbidities admitted to Changsha Public Health Center because of COVID-19. During hospitalization, she developed co-bacterial infection and acute respiratory distress syndrome, and received broad-spectrum antibacterial therapy, invasive mechanical ventilation and extracorporeal membrane oxygenation. After the acute phase, she developed post-COVID-19 pulmonary fibrosis subsequently treated with pirfenidone. Over 96 weeks after pirfenidone treatment, her modified Medical Research Council Dyspnea level improved to 2 from 4 at discharge. Her 6 minutes walk test distance, total lung capacity, and diffusion capacity for carbon monoxide all increased. Chest CT performed on 2 years after illness onset showed regressing fibrosis. The Hospital Anxiety and Depression Scale, Athens Insomnia Scale, and 36-Item Short Form Health Survey questionnaire all improved.

**Conclusion:**

Post-COVID-19 pulmonary fibrosis is a challenging consequence of COVID-19, and our case suggests that pirfenidone may be an effective treatment option.

## Introduction

Since the first report of the Coronavirus disease 2019 (COVID-19) caused by the Severe Acute Respiratory Syndrome Coronavirus 2 (SARS-CoV-2) in Wuhan city on December 2019, the COVID-19 pandemic has lasted more than 2 years. As of March 29, 2022, more than 480 million confirmed cases and 6 million deaths had been reported worldwide according to the World Health Organization (WHO) (1). The clinical manifestations, pathophysiology, and treatment during the acute phase of COVID-19 are well described (2, 3). Several long-term longitudinal follow-up cohorts of patients recovered from COVID-19 have shown that although most patients recover well, up to 57.7–100% of patients still have persistent lung structural abnormalities and pulmonary function impairment (4–6). The common pattern of pulmonary interstitial lesions, also known as post-COVID-19 pulmonary fibrosis or COVID-19 pulmonary fibrosis-like (7), were ground glass opacities (GGOs), ground-glass attenuation and reticular abnormalities (8). At the same time, chest imaging showed bilateral diffuse interstitial lung disease among patients who complicated acute respiratory distress syndrome (ARDS) during acute phase (9). Relatedly, these survivals suffered from more serious ‘Long COVID' (10, 11), a condition described by signs and symptoms of COVID-19 that last longer than 12 weeks and are not explained by an alternative diagnosis, with the most common symptoms of dyspnea, fatigue and ‘brain fog'. However, there was no definite long-term medical advice or treatment to reverse pulmonary fibrosis for this population.

Pirfenidone, one of only two drugs approved for the treatment of idiopathic pulmonary fibrosis (IPF) in the world, is a class of pleiotropic pyridine compounds with anti-inflammatory, anti-fibrotic and antioxidant properties. Another drug is nintedanib, which can significantly reduce the absolute value of the drop in forced vital capacity (FVC) among IPF patients (12). The results of a multi-center, double-blind, randomized controlled phase 3 clinical study (ASCEND study) suggest that pirfenidone significantly delayed the decline in FVC in patients with IPF at 52 weeks compared with placebo (−235 ml vs. −428 ml, *P* < 0.001) (13). Besides, at 52 weeks, pirfenidone can reduce all-cause mortality in patients with IPF according to a pooled analyse and meta-analyses (14). To our knowledge, the effectiveness of pirfenidone in the treatment of post-COVID-19 pulmonary fibrosis is unclear. Here, we report a case of post-COVID-19 pulmonary fibrosis treated with pirfenidone over 96 weeks. During the 2-year follow-up period, the patient's symptoms, diffusing function, and interstitial lesions showed continuous improvement.

## Case Description

**Figure 1:**
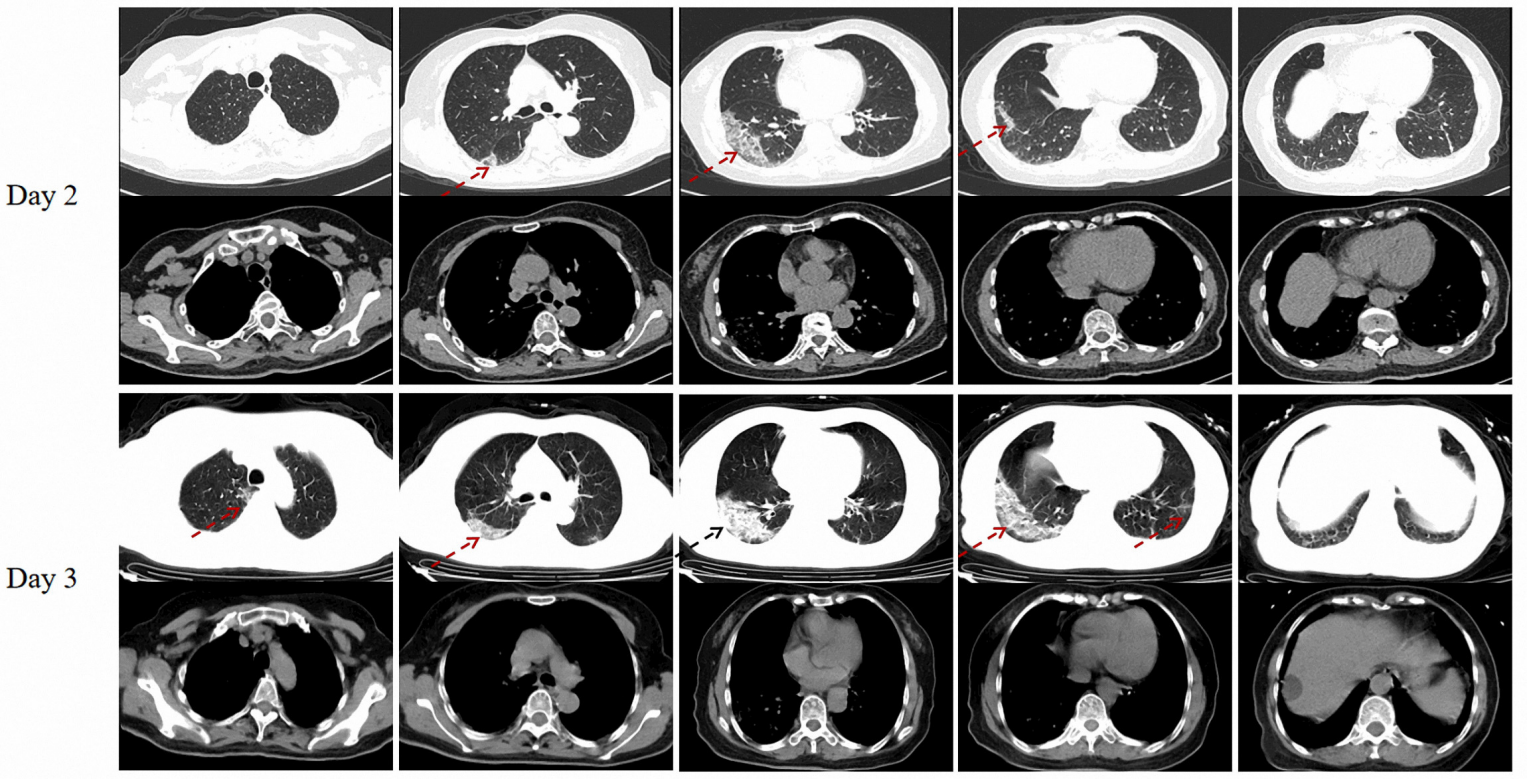
Chest CT of the patient on day 2 and day 3 (On admission) after illness onset. Red dotted arrow: multiple ground glass opacities (GGOs); Black dotted arrow: consolidation.

A previously healthy, 66-year-old female patient presented to the emergency department of Xiangya Hospital on February 2, 2020 (day 2 of illness onset) with a 1-day history of dizziness, nausea and diarrhea. There were no background of pulmonary problems and impaired physical capacity. She underwent a quantitative Real-time Reverse Transcription-Polymerase Chain Reaction (RT-PCR) test for SARS-CoV-2 and a chest computed tomography (CT) examination because of a travel history from Wuhan City within 14 days. The results showed positive to SARS-CoV-2 and multiple GGOs in the right lower lobe (Figure 1). Consequently, she was referred to the Changsha Public Health Center for isolation and hospitalization (day 3).

On admission, physical examination showed a body temperature of 37.3°C, a level at 98% of resting finger oxygen saturation without additional oxygen supplement, overweight with a body mass index (BMI) of 25.3 kg/m^2^, and moist rales in bilateral lower lungs. The laboratory examination results were as follows: white blood cell (WBC) count was 2.79 × 10^9^/L, with a lymphocyte percentage of 22.2%, the procalcitonin concentration was 0.054 ng/mL, the level of C-reactive protein was 65.7 mg/L, the erythrocyte sedimentation rate was 106 mm/h, the ferritin level was 505.5 ng/mL. The serum β-(1,3)-glucan, galactomannan, RT-PCR results for influenza A and B viruses were normal. A reviewed chest high resolution CT taken on February 3 (day3) presented new and progressive, diffuse, peripheral GGOs in the right upper lobe, right middle lobe and left lower lobe, and consolidation in the right lower lobe (Figure 1), typical of acute COVID-19. She was diagnosed with COVID-19 (common type) according to the Chinese Clinical Guidance for COVID-19 Pneumonia Diagnosis and Treatment (4th edition) (15), and received oral antiviral drugs Arbidol and low dose intravenous methylprednisolone hemisuccinate. From day 9, she developed progressive hypoxemia due to suspected co-bacterial infection and COVID-19 aggravation. She was treated with empirical broad-spectrum antibiotics (moxifloxacin, piperacillin tazobactam, meropenem), intravenous immunoglobulin convalescent serum and supported by non-invasive high concentration oxygen. On day 31, she became critically ill and invasive mechanical ventilation (IMV) was conducted because of refractory hypoxemia and progressive lung shadows. Immediately, she was commenced on extracorporeal membrane oxygenation (ECMO) on the next day after IMV. The antibiotics were adjusted to polymyxin B sulfate combined with linezolid, based on a report of bronchoalveolar lavage fluid metagenomic next-generation sequencing which detected 2,55,418 sequences mapped to *Actinomyces odontolyticus* and 3,897 sequences mapped to *Staphylococcus aureus*. Her hypoxia was improved on day 37. She was weaned off ECMO support, meanwhile, de-escalation of antibiotic therapy was conducted. On day 89, she was extubated and freed from IMV.

**Figure 2:**
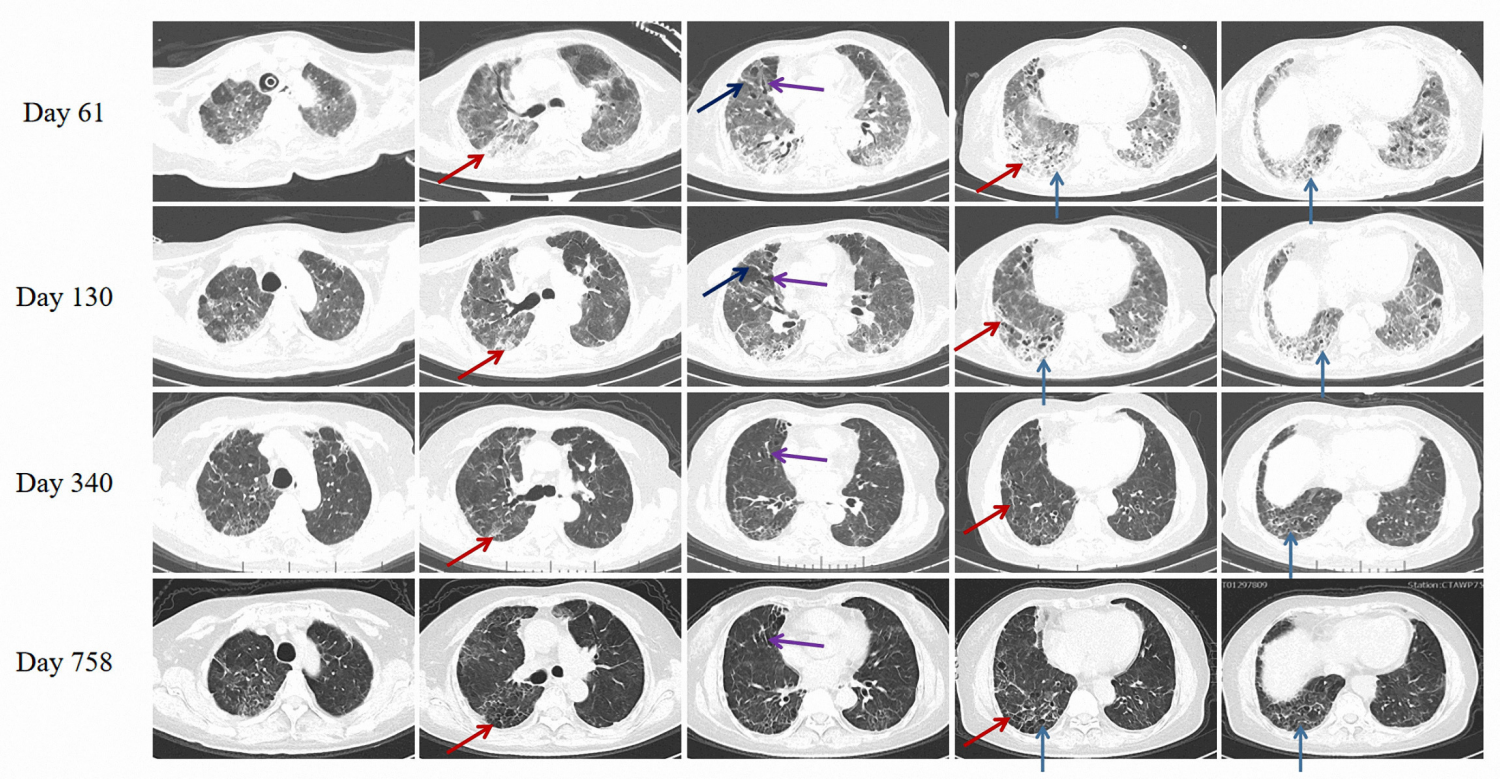
Chest CT on day 61 (Starting taking pirfenidone), day 130 (At discharge), day 340 and day 758 after illness onset. Red solid arrows: subpleural reticular abnormalities and fibrous cords. Black solid arrow: interlobular septal thickening. Purple solid arrow: traction bronchiectasis. Blue solid arrow: honeycomb-like change under the subpleura.

Chest CT scanned on day 61 showed diffuse interstitial changes, with extensive areas of subpleural and peribronchovascular septal thickening, subpleural reticular abnormalities, traction bronchiectasis, and honeycomb-like changes under the subpleura in both lung fields, which manifested as interstitial lung disease (ILD) (Figure 2), and she started taking pirfenidone (Initially 600 mg per day, after 1 week maintain 1,800 mg per day). After 2 months of therapy, her dyspnea and pulmonary fibrosis improved (Figure 2), and subsequently, the patient was discharged on day 130, with a level of modified Medical Research Council Dyspnea Scale (mMRC) at 4 (Table 1). The patient maintained long-term pirfenidone therapy at home without any liver function damage and hematological complications, and she was followed up on the 7th (1 year after onset) and 20th (2 year after onset) month after discharge. The patient's symptoms and physical capacity had improved with the mMRC scores improved from 4 to 2 and 6-min walking test distance (6-MWD) recovered to 309 meters from 188 meters during treatment. Similarly, the Hospital Anxiety and Depression Scale (HADS), Athens Insomnia Scale (AIS), and Chinese vision of the 36-Item Short Form Health Survey questionnaire (SF-36) all improved on 2 year after initial insult. Pulmonary function tests showed improvement in restrictive ventilatory and diffusing function, with FVC improved from 1.98 liters to 2.30 liters, total lung capacity (TLC) improved from 61.7% of predict to 72.3% and diffusion capacity for carbon monoxide (DLCO) improved from 30.3% of predict to 47.9%. Reticular abnormalities, interlobular septal thickening and honeycomb-like changes were reduced in bilateral lung fields on chest CT screened on day 758 (Figure 2). The patient gradually returned to activities of daily living, with self-care on the 7th month and physical activity on the 20th month after discharge.

**Table 1:** Evolution of clinical features from illness onset and taking pirfenidone (2 months after onset).

**Clinical features**	**Before COVID-19**	**Acute stage**	**At discharge (4 months after onset)**	**1 year after onset**	**2 years after onset**
**Symptoms**					
Fever		√			
Cough		√	√	√	√
Dyspnoea		√	√	√	√
Headache		√			
Fatigue		√	√	√	√
Myalgia		√	√	√	
Nausea/Vomiting		√			
Diarrhea		√			
Chest pain		√	√	√	√
Anosmia		√	√		
Dysgeusia		√	√		
Other		√	√		
Pulmonary rale	/	Moist rale	Velcro rale	Velcro rale	Velcro rale
mMRC, level	0	/	4	3	2
6-MWD, m	/	/	/^*^	188	309
**HADS score**					
Anxiety	/	/	/	1	0
Depression	/	/	/	0	0
AIS Score	/	/	/	10	2
**SF-36 score**					
Physical functioning	/	/	/	25	85
Role-physical	/	/	/	0	100
Bodily pain	/	/	/	72	74
General health	/	/	/	35	55
Vitality	/	/	/	55	85
Social functioning	/	/	/	37.5	87.5
Role-emotional	/	/	/	100	100
Mental health	/	/	/	84	92
Health transition	/	/	/	75	75
**Pulmonary function**					
FVC, L	/	/	/^*^	1.98	2.30
FEV1, L	/	/	/^*^	1.64	1.80
FEV1/FVC	/	/	/^*^	0.82	0.83
TLC, % of predict	/	/	/^*^	61.7	72.3
DLCO, % of predict	/	/	/^*^	30.3	47.9

* *The patient can't tolerate it. mMRC, modified Medical Research Council Dyspnea Scale; 6-MWD, 6 Minutes Walk Test Distance; HADS, Hospital Anxiety and Depression Scale; AIS, Athens Insomnia Scale; SF-36, Chinese vision of the 36-Item Short Form Health Survey questionnaire; FVC, Forced Vital Capacity; FEV1, Forced Expiratory Volume in 1 s; TLC, Total Lung Capacity; DLCO, Diffusion Capacity for Carbon monoxide*.

## Discussion

The developing and toughest challenge is how to manage COVID-19 sequelae, in view of our greater understanding of the consequences of post-COVID-19 infection (11). A cohort study of 2,73,168 discharged patients from the United Kingdom showed that 36.5% of patients recorded one or more symptoms between 3 and 6 months after discharge (16).

Post-COVID-19 pulmonary fibrosis is a heterogeneous disease with different imaging performance. The researchers found that residual changes after acute stage vary over time. A retrospective study shown that (17), 3 months after acute phase, pulmonary fibrosis was clinically confirmed in 56% and 71% of the patients with moderate and severe COVID-19 symptoms respectively. In a prospective study (4), Wu et al. found that 78% of patients (65/83) had residual changes on CT at 3 months after discharge and the most common CT features were GGO (78%), interlobular septal thickening (34%), reticular opacity (33%), and subpleural curvilinear opacity (11%). In the same study, 24% of patients (20/83) had persistent imaging abnormalities during the 1-year follow-up, without progressive interstitial changes and definite intervention. However, another prospective study presented higher proportion. According to Huang and his colleagues (6), the rate of persisting imaging abnormalities at 12 months after discharged was 76% among patients with severity scale 5 to 6. The phenomenon was similar to severe acute respiratory syndrome (SARS) and Middle East respiratory syndrome (MERS) survivors. In a retrospective study (18), 21.5% (67/311) of SARS patients had radiographic pulmonary fibrosis at discharge and 40 patients exhibited improvement of fibrosis at one-year follow-up. Analogously, a study of MERS (19) survivors revealed 33% of patients presented chest radiological abnormalities at 80 days after discharge.

In our case, the patient presented with marked restrictive ventilatory dysfunction and impaired diffusing function, which is consistent with previous studies. Mo et al. (20) reported abnormal diffusion function and decreased TLC in 47.2 and 25.0% of patients at discharge. Although this percentage was undoubtedly underestimated, disease severity in the acute phase was inextricably linked to pulmonary function at discharge. Significant difference in impaired diffusing capacity among the different groups of severity, which accounted for 30.4% in mild and 84.2% in severe illness, respectively (20). Meanwhile, the proportion of anomalies gradually decreased over time. Follow-up studies revealed that the proportion of survivors with abnormal pulmonary diffusing function decreased to 35–55% and 21–33% at 3 and 12 months after discharge respectively (4, 6, 21). In a 12-month longitudinal follow-up study, GGO and irregular lines were positively associated with risk of lung diffusion impairment at 12 months (6). This suggested that further reductions in pulmonary fibrosis could improve their lung function and quality of life.

The treatment strategy, oral pirfenidone, 1,800 mg per day (22), for post-COVID-19 pulmonary fibrosis, was bold, novel and aggressive, given there was no reliable evidence of effectiveness of pirfenidone in this population. This protocol was developed because her chest imaging changes were consistent with ILD, including common IPF. In the CAPACITY-004 study (23), pirfenidone reduced decline in FVC with a mean FVC change at −8.0% in the pirfenidone (2,403 mg/day) group and −12.4% in the placebo group at week 72. However, in another concurrent RCT study (CAPACITY-006), the difference between groups in FVC change at week 72 was not significant. More attention should be paid to the application of pirfenidone in interstitial lung diseases other than IPF. A multi-center, double-blind, randomized controlled phase 2 clinical study (uILD study) (24) reported that, for unclassifiable interstitial pneumonia, the use of pirfenidone (2,403 mg/d) for 24 weeks significantly delayed the decline in FVC (−17.8 vs. −113 ml, *P* = 0.002). Although evidence shown that pirfenidone could alleviate inflammatory responses in hospitalized adult patients with severe COVID-19 (25), its effectiveness in post-COVID-19 pulmonary fibrosis is still unknown.

Surprisingly, encouraging effectiveness was observed in our patient. Fibrotic lesions gradually subsided, lung diffusing function improved and she returned to normal daily life after pirfenidone therapy. However, this study has some limitations. First, there is reporting bias and it was unclear whether her improvement was benefited from pirfenidone or a natural regression of the disease course. Second, this study is not general, and the effect of pirfenidone in patients with other lung diseases is still unknown. Ongoing clinical trials (26–29) about pirfenidone and deupirfenidone in the treatment of post-COVID-19 pulmonary fibrosis may be able to provide a clear answer (Table 2).

**Table 2:** Clinical trials^a^ of pirfenidone and deupirfenidone^b^ for the treatment of post-COVID-19 pulmonary fibrosis.

**Identifier**		**Start time**	**Study design**	**Phase**	**Objects**	**Number enrolled and distribution**	**Intervention**	**Primary outcome**
NCT04607928 (26)		29-Oct-20	Multicenter, randomized, placebo-controlled	II	Patients with fibrotic lung sequelae after recovery from acute phase of severe COVID pneumonia	148 2:1 (Pirfenidone: Placebo)	Pirfenidone: 2403 mg/day for 24weeks; Controlled: placebo for 24 weeks;	Change from baseline in % in forced vital capacity (FVC) and % fibrosis in high resolution computed tomography of the lung
NCT04856111 (27)		22-Apr-21	Singlecenter, randomized	IV	Patients with fibrotic lung disease after COVID-19	48 1:1 (Pirfenidone: Nintedanib)	Pirfenidone: 2,400 mg/day for 24 weeks; Nintedanib: 300 mg/day for 24 weeks;	Change in the forced vital capacity (FVC)
NCT04652518 (28)	Part A	3-Dec-20	Multicenter, randomized, double-blind, placebo-controlled	II	Adults with post-acute COVID-19 respiratory complications	168 1:1 (Deupirfenidone: Placebo)	Deupirfenidone: 500 mg for 91 days; Controlled: placebo for 91 days	Change in distance walked on the 6-minutes walk test (6 MWT)
	Part B	-	Open label extension study	II	Eligible patients who completed part A	-	Deupirfenidone: an additional 91 days	Assess the longer-term safety, tolerability, and efficacy of Deupirfenidone through up to 182 days of treatment; (One of the secondary objective: Inflammatory, fibrosis and other biomarkers;)
ChiCTR2000030892 (29)		16-Mar-20	Parallel	-	Patients with severe post-COVID-19 fibrosis	40 1:1 (Pirfenidone: blank control)	-	Change in HRCT pulmonary fibrosis score

a *Data retrieved from ClinicalTrials.gov and chictr.org;*

b *Deupirfenidone: a selectively deuterated form of pirfenidone*.

## Conclusion

In summary, our study reported a case of post-COVID-19 pulmonary fibrosis treated with long-term oral pirfenidone and the patient recovered well in terms of symptoms, pulmonary function and chest CT images. Pirfenidone may be an effective therapeutic strategy for post-COVID-19 pulmonary fibrosis. RCTs are needed for validation.

## Data Availability Statement

The original contributions presented in the study are included in the article/supplementary material, further inquiries can be directed to the corresponding author/s.

## Ethics Statement

The studies involving human participants were reviewed and approved by the Review Board of the Second Xiangya Hospital of Central South University. The patients/participants provided their written informed consent to participate in this study.

## Author Contributions

XZ and DY collected the data and wrote the manuscript. XK, CW, SQ, LW, YL, and ZY analyzed, interpreted the data, and performed the clinical assessment. ZZ and HL designed the study and performed the clinical assessment. All authors reviewed, edited, and approved the final manuscript.

## Funding

This study was supported by the National Natural Science Foundation of China (82070003 to HL), Natural Science Foundation of Hunan Province, China (2021JJ30943 to HL), the Science and Technology Program of Changsha, China (kq1901120 to HL), Xiangya Clinical Big Data System Construction Project in Pulmonary Inflammatory Disease of Central South University, and the National Key Clinical Specialty Construction Projects of China.

## Conflict of Interest

The authors declare that the research was conducted in the absence of any commercial or financial relationships that could be construed as a potential conflict of interest.

## Publisher's Note

All claims expressed in this article are solely those of the authors and do not necessarily represent those of their affiliated organizations, or those of the publisher, the editors and the reviewers. Any product that may be evaluated in this article, or claim that may be made by its manufacturer, is not guaranteed or endorsed by the publisher.
